# Phagostimulant bait sprays improve control of spotted wing drosophila (*Drosophila suzukii*) in soft fruit

**DOI:** 10.1007/s10340-025-01925-y

**Published:** 2025-08-02

**Authors:** Ralph Noble, Adam Walker, Greg Deakin, Andreja Dobrovin-Pennington, Bethan Shaw, Sebastian Hemer, Michelle T. Fountain

**Affiliations:** 1 Microbiotech Ltd, Pershore College, Pershore, Worcestershire UK; 2 Niab, East Malling, Kent, UK

**Keywords:** Berries, Entomopathogenic fungus, Insecticide, Integrated pest management, Pesticide, Spotted wing drosophila

## Abstract

By attracting and stimulating feeding on spray droplets, phagostimulant baits provide an opportunity to increase the efficacy of crop protection products against the spotted wing drosophila (*Drosophila suzukii*). Here, we examined the use of a high-sugar, plant-derived bait (ProBandz^®^, PB) in combination with low dose insecticides and an entomopathogenic fungus *Metarhizium anisopliae* strain 35.79 for control of *D. suzukii.* We compared the efficacy of treatments in laboratory jar bioassays and in semi-field strawberry experiments using laboratory *D. suzukii* cultures, and in field strawberry and raspberry experiments on natural *D. suzukii* infestations. *M. anisopliae* 35.79 increased *D. suzukii* mortality in jar bioassays but did not affect oviposition. There was no evidence that combining *M. anisopliae* 35.79 with PB led to increased efficacy, and in a semi-field experiment this combination led to an increase in *D. suzukii* larvae in fruit. Deltamethrin in PB droplets was effective in increasing mortality and reducing oviposition in jar bioassays but deltamethrin + PB bait sprays were ineffective in a field raspberry experiment. PB increased the *D. suzukii* control efficacy of lambda-cyhalothrin in jar bioassays. Low volume bait sprays with 8% of the full field rate of lambda-cyhalothrin in semi-field and field strawberry experiments were as effective in controlling *D. suzukii* as full rate, high volume insecticide sprays but without causing pesticides residues in the fruit. This work will provide evidence supporting the reduction of dependence and risk of resistance to the two main insecticides used for *D. suzukii* control: spinosad and cyantraniliprole.

## Introduction

The spotted wing drosophila (*Drosophila suzukii* (Matsumura) Diptera: Drosophilidae) is a globally significant pest of fruit crops. Bait sprays, which involve the use of phagostimulants and odorants to promote *D. suzukii* attraction and a feeding response to applied insecticides, can be an important control method (Tait et al. 2021). Ingested insecticides applied in bait sprays are effective at lower doses than are required in full field rate applications that rely on random and external pest contact (Noble et al. 2022; Babu et al. 2023). Pesticide impacts on beneficial organisms and non-target organisms such as bees are therefore minimised (Fountain et al. 2025) and the risk of residues in fruit reduced (Noble et al. 2021, 2022). Suitable *D. suzukii* baits usually include sugar and protein sources, as well as odorants associated with ripening or fermenting fruit (Noble et al. 2021). ProBandz^®^ (PB) (Russell IPM Ltd, Deeside, Flintshire, UK) is a plant-derived, high sugar (54%w/w) phagostimulant bait that is approved in the UK as an adjuvant with authorised plant protection products on edible and non-edible crops (UK Adjuvant Number A0943).

A limited number of pesticides can give effective control of *D. suzukii* and growers have become reliant on their use (Shaw et al. 2019a; Shawer et al. 2019). Due to *D. suzukii* control efficacy and limited impact on several beneficial organisms, the two main insecticides used and authorised for *D. suzukii* control in fruit crops in the UK are spinosad and cyantraniliprole (Shaw et al. 2019a). However, repeated applications of spinosad have resulted in resistance of *D. suzukii* (Gress and Zalom 2019) and exposure to cyantraniliprole sprays has increased tolerance of *D. suzukii* (Shaw 2021). *Ganaspis kimorum* Buffington (Hymenoptera: Figitidae) is a promising biological control agent against *D. suzukii*, and these compounds have been shown to be toxic towards this natural enemy (Fellin et al. 2024; Stahl et al. 2024). Although synthetic pyrethroid insecticides such as deltamethrin and lambda-cyhalothrin provide *D. suzukii* control, they also have indiscriminate impacts on beneficial organisms, such as the parasitoid wasps *Trichopria drosophilae* Perkins (Hymenoptera: Diapriidae) (Gao et al. 2024) and *G. kimorum* (Fellin et al. 2024). However, laboratory and small-scale cage *D. suzukii* experiments have shown promising results with reduced doses of synthetic pyrethroids in bait sprays (Helsen and van der Sluis 2017; Noble et al. 2019) although field-scale experiments are lacking. Previous work on the use of bait sprays for *D. suzukii* control has concentrated on the use of reduced doses of spinosad and cyantraniliprole (Noble et al. 2022; Babu et al. 2023), as well as products that are no-longer authorised for use in the UK, such as acetamiprid and malathion (Fanning et al. 2021).

Entomopathogenic fungi (EPF) have been investigated as a biological control for *D. suzukii* and an alternative to chemical pesticides. Lee et al. (2019) found some *D. suzukii* control efficacy of commercially available strains of the EPF species *Beauveria bassiana*, *Metarhizium brunneum* and *Isaria fumosorosae*. Furuie et al. (2022) found that *B. bassiana* was ineffective in killing *D. suzukii* but Galland et al. (2023) found it caused 95% mortality but only after a 10 day period; four other EPFs, including *Metarhizium anisopliae*, *M. brunneum* and *I. fumosorosae* were ineffective. In other screening tests (Russell IPM, unpublished) a strain of *M. anisopliae* (35.79) produced 90% *D. suzukii* mortality, 12 days post-inoculation, and was more effective than two other *M. anisopliae* strains, as well as *M. brunneum*, *B. bassiana* and *I. fumosorosae*. This previous work on EPF efficacy was conducted in laboratory bioassays; information on field efficacy of EPF in *D. suzukii* control is lacking. In addition, oral ingestion of *B. bassiana* and *M. anisopliae* killed adults of the grain storage beetle pest *Sitophilus granaries* (Batta 2018) but the use of EPFs in bait sprays has not been investigated.

Aims of this work were to (1) examine the use of *M. anisopliae* 35.79 with PB for control of *D. suzukii;* (2) compare the *D. suzukii* control efficacy of full rate insecticide, including synthetic pyrethroid, sprays with low-rate insecticide applications in PB bait sprays; and (3) compare the efficacy of PB, with EPF or reduced dose insecticides, on *D. suzukii* mortality in jar bioassays with that measured in semi-field experiments.

## Materials and methods

### Bait, insecticides, metarhizium inoculum and *Drosophila suzukii* colony

PB solution was prepared by mixing 5% v/v PB in warm (50ºC) tap water. The following insecticides, with concentrations of active ingredients shown in Table 1, were used: cyantraniliprole (products Benevia^®^ and Exirel^®^, Du Pont, Wilmington, DE, USA), deltamethrin (Decis ProTech^®^, Bayer Crop Science, Cambridge, UK), lambda-cyhalothrin (Hallmark Zeon^®^, Syngenta, Basel, Switzerland) and spinosad (Tracer^®^, Dow AgroSciences, Zionsville, IA, USA). For cyantraniliprole, the Exirel product was used for laboratory bioassays and raspberry crop field application and the Benevia product was used for strawberry crop field application according to the UK authorisations for these products. A granular solid inoculum of *M. anisopliae* 35.79 containing 3.4 – 6.8 × 10^7^ cfu g^−1^ was prepared by Russell IPM. *M. anisopliae* 35.79 inoculum suspensions were prepared by adding and shaking 11 g solid inoculum in one litre of tap water or PB solution containing 0.2 ml of a detergent (Biocoat C, Russell IPM) to aid spore dispersal.

**Table 1:** Concentrations of active ingredient (a.i.) in the insecticide products and those used in jar bioassays, and in the low and full rate sprays in the semi-field and field scale strawberry and raspberry experiments, together with the corresponding field application rates

Active ingredient	Product, a.i. (g l^−1^)	Concentration of a.i. (mg l^−1^) in					Field rate of a.i. (g ha^−1^) in			
		Bioassay	Strawberry		Raspberry		Strawberry		Raspberry	
		(number)	Low	Full	Low	Full	Low^a^	Full^b^	Low^a^	Full^b^
Cyantraniliprole	Exirel^®^, 100	12.0 (1,2)	–	–	90.0	180	–	–	3.6	90.0
Cyantraniliprole	Benevia^®^, 100	–	90.0	150	–	–	3.6	75.0	–	–
Deltamethrin	Decis ProTech^®^, 15	8.3 (2)	8.3	10.0	8.3	–	0.3	5.0	0.3	5.0
Lambda-cyhalothrin	Hallmark Zeon^®^, 190	19.0 (2)	36.1	28.5	–	–	1.4	14.3	–	–
Spinosad	Tracer^®^, 480	3.4 (2)	96.0	144	96.0	192	3.8	72.0	3.8	96.0

^a^Applied at 40 l ha^−1^, ^b^applied at 500 l ha^−1^

The *D. suzukii* culture used in the jar bioassays and semi-field experiments was obtained near Trentino, Italy in 2013 and reared in cages at 20 °C in a 12:12 L:D photoperiod on a diet of dried and rewetted Drosophila medium with added yeast (Philip Harris Education, Hyde, Cheshire). The cultures were discarded and renewed when the oldest flies were 14 days old and no new wild flies were added.

### Jar bioassays

A jar bioassay (Noble et al. 2019) was used to test the effect of insecticides at sub-lethal concentrations, and *M. anisopliae* 35.79, on *D. suzukii* mortality and oviposition. Sub-lethal concentrations of insecticides, whereby 33% of exposed adult individuals did not survive a three-day exposure in the jar bioassay described below (LC_33_), were determined from replicated range-finding tests (data not shown). Briefly, the bioassay involved applying with a micropipette, six 10 µl droplets of low dose insecticides or *M. anisopliae* 35.79 inoculum, with or without PB bait, to wild blackberry leaves sourced from hedgerows. Two such leaves, and a leaf with six 10 µl sugar solution droplets, were placed in ventilated clear plastic jars lined with a moist filter paper in the base. The droplets were first dried for one hour before placing the leaves in the jars which also contained a small Petri dish of a grape juice agar oviposition medium (Noble et al. 2019). This was prepared from agar (No. 3, 34.7 g, Oxoid Ltd, Basingstoke, Hants.), red grape juice (333 ml, Sainsbury’s Supermarkets Ltd, London), dextrose (33.3 g, Oxoid Ltd) and Nipagin (2.0 g, Sigma-Aldrich, Gillingham, Dorset, UK) per litre distilled water. Seven mated female and five male 4—6 day old summer morph *D. suzukii* adults were introduced in each jar (flies 3—14 days old were used for preliminary range-finding tests but this did not significantly influence the mortality effects of the insecticide concentration treatments). The food source plates were removed from the fly colonies 12 h before the start of the bioassays. The jars were kept at 21 ± 1 °C with a photoperiod of 16:8 (L:D). The number of eggs in the oviposition medium was counted after three days and fly mortality at 2—4 day intervals for up to 14 days depending on the bioassay. Any larvae that hatched during this period were added to the egg counts.

In Bioassay 1, the insecticide or inoculum treatments applied as droplets in water or 5% v/v PB solution to leaves were (i) untreated control, (ii) cyantraniliprole, product details and concentration in Table 1, or (iii) *M. anisopliae* 35.79 suspension containing 6.3 × 10^5^ cfu ml^−1^. There were 3 insecticide or inoculum treatments × 2 bait treatments (water or bait solution) × 10 replicates, hence 60 test jars. Two additional replicate jars were prepared of treatments (i) and (iii) in water and PB solution to determine the survival of *M. anisopliae* 35.79 inoculum in the dried droplets on the leaves at 2—3 day intervals for 9 days. Leaves were washed with 1 ml sterile distilled water and 5 µl of the washings were plated on potato dextrose agar + chlortetracycline (Oxoid Ltd, Basingstoke, Hants., UK), incubated at 20 °C for one week to count colony forming units of *M. anisopliae.*

In Bioassay 2, the insecticide treatments applied as droplets in water or 5% v/v PB solution to leaves were (i) untreated control, (ii) cyantraniliprole, (iii) spinosad, (iv) lambda-cyhalothrin, or (v) deltamethrin. Insecticide product details and concentrations are in Table 1. There were 5 insecticide treatments × 2 bait treatments (water or bait solution) × 4 replicates, hence 40 test jars.

### Semi-field scale strawberry experiments

Experiments were conducted at Niab, East Malling in small cropping tunnel compartments covered with fine mesh to limit entry or exit of *D. suzukii* (Noble et al. 2021). The roofs and upper sides of the tunnels were covered with standard commercial polythene cladding leaving the ends of the tunnels and the lower 1 m of the side walls covered only in mesh. Floors of the tunnels were covered with Mypex membrane sheeting. Ever-bearer cultivar strawberry plants were grown in coir grow bags, each containing eight plants. The positioning and number of grow bags per tunnel varied between experiments. Insecticide applications at the full field rates were made with a motorised, air assisted knapsack sprayer with a high water volume, equivalent to 500 l ha^−1^ (Noble et al. 2021). Low-rate insecticide applications were made using a motorised knapsack sprayer with PB and a water volume equivalent to 40 l ha^−1^ (Table 1). Other details of application of full field rate with high volume sprays and low rate and volume bait sprays, strawberry crop husbandry and environment monitoring are the same as in Noble et al. (2021).

In each compartment 10 female and 10 male adult summer morph 3—14 day old *D. suzukii* were introduced one day after the first, second and third sprays (30 female and 30 male adults were added to each compartment in total). Samples of 20 ripe strawberries were picked one day before the first spray and six days after each of the four sprays from each compartment. The samples were incubated for two days at 20 °C; larvae were then extracted using the sugar flotation method in Shaw et al. (2019b). No larvae were present in fruit samples taken before sprays were applied.

Air temperature and relative humidity (RH) among the strawberry plant canopies were recorded by sensors (type HMP 31 UT, Vaisala, Vantaa, Finland) and data loggers (Grant Instruments, Cambridge). Average daily maximum and minimum temperatures were 26 ºC and 15 ºC in Experiment 1 and 26 ºC and 12 ºC in Experiment 2. Average daily maximum and minimum RH was 94% and 59% in Experiment 1 and 98% and 54% in Experiment 2.

#### Semi-field scale strawberry Experiment 1

Two adjacent rows of five coir grow bags were positioned on 10 cm height plastic crates in every compartment. The strawberry plant cropping area in each compartment measured 5.0 × 0.8 m. Strawberry plants cv. Matilda were first sprayed on 6 July 2022 when some of the plants in all the compartments had almost ripe fruit, most of the plants had fruit that was showing pink flush, and all plants had fruit that was at white fruit stage. The plants were then sprayed three further times, with alternating weekly sprays of spinosad (product Tracer^®^) and cyantraniliprole (product Benevia^®^) at the full field rates and high volume (treatment 1) or at low rates with PB at low volume (treatment 2) (Table 1). A suspension of *M. anisopliae* 35.79 containing 3.3–7.0 × 10^5^ cfu ml^−1^ in PB solution was applied at low volume (treatment 3). Control plots remained unsprayed (treatment 4). Strawberry leaves from two replicate compartments of treatments 3 and 4 were sampled and tested for *Metarhizium* cfu as previously described, immediately after each spraying and again after 2, 5 and 7 days. There were five replicate tunnel compartments of each treatment arranged in a randomised block design.

#### Semi-field scale strawberry Experiment 2

Five coir grow bags were set out in a single row on a 1.5 m high table-top growing system in every compartment. The strawberry plant cropping area in each compartment measured 5.0 × 0.5 m.

Strawberry plants cv. Malling Supreme were first sprayed on 18 June 2024 at white fruit stage, and then three further times with alternating weekly sprays of lambda-cyhalothrin (product Hallmark Zeon^®^) and deltamethrin (product Decis ProTech^®^) at the full field rates (treatment 1) or at low rates with PB (treatment 2) (Table 1). Plants were also sprayed four times with alternating weekly sprays of spinosad (product Tracer^®^) and cyantraniliprole (product Benevia^®^) at low rates with PB (treatment 3) (Table 1). Control plots remained unsprayed (treatment 4). There were five replicate compartments of each treatment arranged in a randomised block design. Data of a fifth insecticide treatment, which was part of the same experiment blocking structure, were included in the statistical analysis, thereby improving estimation of the effect of blocks, but the treatment results are not presented due to confidentiality.

Samples of strawberries for pesticide residue analysis were taken immediately after each of the spray applications. Pooled, 1 kg samples of fruit from all replicates of treatments were analysed by Tentamus QTS Analytical Ltd, Sittingbourne, Kent, UK using liquid chromatography-mass spectrometry (LC–MS) (ISO 9001 certification). The detection limit for pesticide residues was 0.01 mg kg^−1^ fruit.

### Field scale experiments

Experiments were conducted in commercial crops in 48—163 m length and 6.9 m width span polytunnels. Primocane raspberry plants were potted with three canes in 7.5-L pots containing a coir growing medium and arranged in two rows 3 m apart, with pots spaced at 0.5 m along the length of the tunnels. Ever-bearer strawberries were grown in raised soil beds covered with polythene; each bed contained two rows of plants spaced 0.5 m apart with plants spaced at 0.5 m along the row. Details of application of full field rate sprays and low rate and volume with bait sprays to strawberries and raspberries are the same as in Noble et al. (2021). The crops were naturally infested with *D. suzukii* from a background population at each site.

Average daily maximum and minimum temperatures in the raspberry plant canopies were 23 ºC and 10 ºC in Experiment 1 and 20 ºC and 12 ºC in Experiment 2. Average daily maximum and minimum RH was 99% and 54% in Experiment 1 and 95% and 62% in Experiment 2.

#### Field scale experiment 1: raspberry

The experiment was conducted in Kent using raspberry cv. Majestic. The tunnel was divided into 8.9 m length compartment plots along the length of tunnel by barriers of insect exclusion mesh (Gromax Industries Ltd, Hadleigh, Suffolk, UK; Gro-Net AA/6, hole size; 0.8 mm × 0.8 mm) which was clipped to the underside of the tunnel hoop structure. Insect exclusion mesh was also deployed along the tunnel leg rows in between the tunnels. The mesh was erected eight days before the first spray and used to reduce free movement of *D. suzukii* between treatment plots and spray drift between compartments. The exclusion mesh was not completely insect proof and allowed some ingress of *D. suzukii* from the surrounding crop. There was a natural population of *D. suzukii* in the crop at the start of the experiment so artificial infestation was not required. Each compartment plot contained 30 pots. Starting at pink flush fruit stage on 7 September 2022, compartments were sprayed with alternating weekly applications of spinosad (product Tracer^®^) and cyantraniliprole (product Exirel^®^), either at full field rate or at low rate with PB (Table 1). Samples of 20 ripe raspberries were picked from each compartment one day before spraying started, six days after each of the four sprays and 13 days after the final spray and assessed for larvae flotation as previously described. There were four replicate compartments of each treatment arranged in a randomised block design. Data of four other insecticide and bait treatments, which was part of the same experiment blocking structure, were included in the statistical analysis, but the results are not presented due to confidentiality.

#### Field scale experiment 2: raspberry

The experiment was conducted in Staffordshire using raspberry cv. Yana in ten adjacent polytunnels. In alternating tunnels, 15 m length plots of 25 pots in the middle of rows were sprayed with a low-rate spray of deltamethrin (product Decis ProTech^®^) with PB (Table 1), on 13 September 2024, seven days before the first harvest. The remaining tunnels were unsprayed. Samples of 30 ripe raspberries were picked from each plot one day before and six days after spraying and assessed for *D. suzukii* adult emergence as described in Noble et al. (2021).

#### Field scale experiment 3: strawberry

The experiment was conducted in Kent using a range of strawberry cultivars in nine adjacent tunnels. Spraying commenced one month after picking had started. On 3 August 2024, five replicate beds were sprayed with either a full field rate and high volume of cyantraniliprole (product Benevia) or a low rate and volume of lambda-cyhalothrin (product Hallmark Zeon) with PB. The same sprays were applied again seven days later. Five control beds remained unsprayed. Samples of 20 ripe strawberries were picked from each plot one day before spraying had started and six days after each spraying and assessed for *D. suzukii* adult emergence as described in Noble et al. (2021).

### Statistical analysis

#### Jar bioassays

Jar bioassay experiments were designed as randomised blocks with the replicates of each factorial set of bait and insecticide/inoculum treatments set up consecutively. In each experiment, an ANOVA was conducted to determine if there were significant main effects or interacting effects of the treatments on mortality or oviposition. The statistical significance of the difference between treatments was determined by conducting two-sided t-tests on means obtained from the ANOVAs. Results were analysed by GenStat Version 13.1 throughout, p ≤ 0.05 was used to determine statistical significance.

#### Semi-field scale experiments

Statistical analysis was carried out separately for each experiment in R 4.1.1 (Anonymous 2021). Analyses used generalised linear mixed models (GLMM) for Experiment 1 or generalised linear models (GLM) for Experiment 2 as appropriate to the experimental design and data. GLMMs were fitted with the glmmTMB package (Brooks et al. 2017). For mixed models, likelihood ratio tests were performed to test for statistical differences between the fixed effects. Post hoc tests were carried out with the emmeans package (Lenth and Emmeans 2021), with p-values corrected for false discovery rate using the Tukey method.

For Experiment 1 a GLMM with negative binomial family was fitted to the data; a random effect model was used due to the repeat measures experimental design. For Experiment 2 a GLM with Poisson family was fitted to the data. Due to complete separation within the data, Firth's Penalized Likelihood method was applied (Lenth and Emmeans 2021). Quasi-likelihood adjustment was made to deviance (for ANOVA) and standard errors (post-hoc tests) to correct for overdispersion.

#### Field-scale experiments

The analyses were conducted in two stages; first to estimate the assessment effect and interaction between assessment and treatment, all data including the pre-treatment assessment was included. Then to estimate the treatment main effect, the model was refitted to the data excluding the pre-treatment assessment. A GLMM with Poisson family was fitted to the data. The model included a random effect of “plot” to account for the non-independence of repeated measurements, and fixed effects for block, assessment, treatment and the interaction between the latter. To enable a reasonable model fit, a Baysian prior was used to estimate treatment × assessment combinations which, though measured, had counts of zero.

## Results

### Jar bioassays

**Fig. 1:**
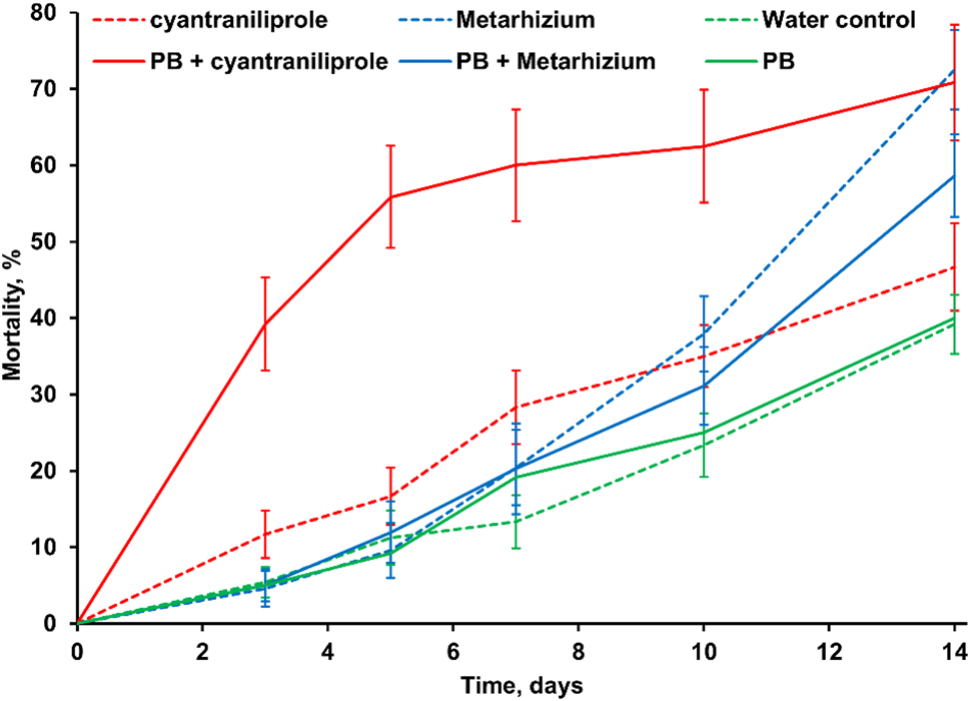
Effect of cyantraniliprole and *Metarhizium anisopliae* 35.79 with and without 5% v/v ProBandz^®^ (PB) on mortality of *Drosophila suzukii* adults in Bioassay 1; mean values, *n* = 10 (± SE)

In Bioassay 1, there were significant effects of insecticide or inoculum (*F*_*2,270*_ = 25.28; *p* < 0.001), PB (*F*_*1,270*_ = 4.02; *p* < 0.001) and insecticide or inoculum × PB × time interaction (*F*_*8,270*_ = 2.96; *p* < 0.001) on *D. suzukii* mortality (Fig. 1). At all time points after the start of the bioassay, mortality was higher in the cyantraniliprole + PB treatment than in the water control (*t*_270_ = 4.85; *p* < 0.001). Cyantraniliprole or PB alone did not significantly affect mortality. Up to day 10 of the bioassay, there was no significant effect of *M. anisopliae* 35.79 on mortality. At day 14, mortality was higher in the *M. anisopliae* 35.79 treatments than in the water control (*t*_270_ = 3.83; *p* < 0.001). There was no significant effect on *D. suzukii* mortality of combining PB with *M. anisopliae* 35.79. Fungal growth typical of *Metarhizium* was observed on 40.4% of the *D. suzukii* cadavers in the *M. anisopliae* 35.79 (treatment iii) in water and PB, but not in any other treatments.

*Metarhizium* counts in leaf washings from treatment (iii) declined from 6.1 (± 2.1) × 10^5^ cfu ml^−1^ at the start of the bioassay to 3.3 (± 1.3) × 10^5^ and 1.5 (± 0.2) × 10^5^ cfu ml^−1^ after 4 and 9 days, respectively. These counts were unaffected by the presence of PB in the droplets applied to the leaves. No *Metarhizium* was detected in the leaf washings from treatment (i) with water only droplets applied.

There was a significant effect of cyantraniliprole × PB interaction on oviposition after 3 days (*F*_*2,54*_ = 6.76; *p* < 0.001). Oviposition per dish was lower in the cyantraniliprole + PB treatment (17 ± 2.9) than in the water control (30 ± 4.8) but was unaffected by the other treatments (combined mean of other treatments 33 ± 4.3).

**Fig. 2:**
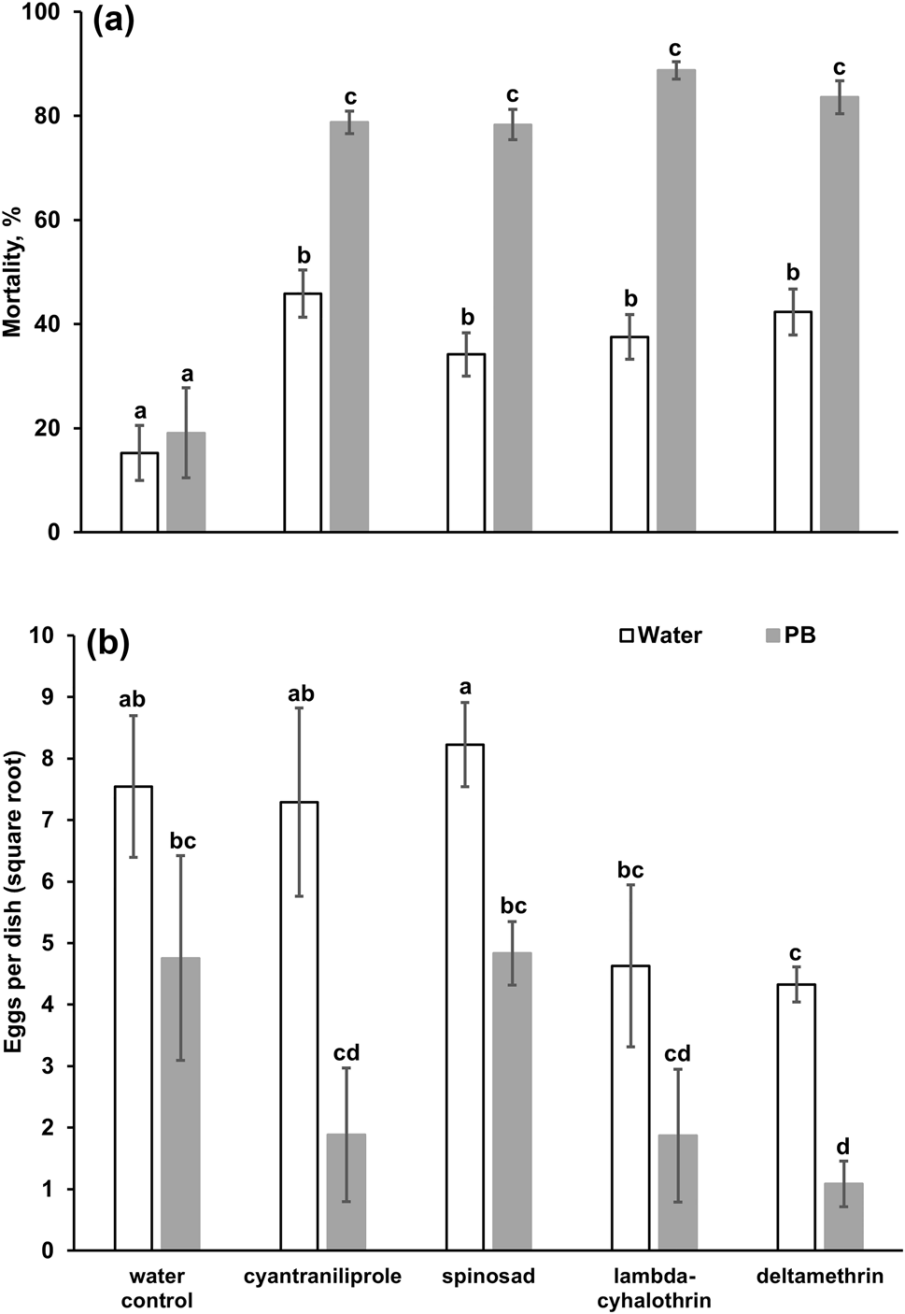
Effect of insecticides and ProBandz^®^ (PB) on **a** mortality and **b** oviposition of *Drosophila suzukii* in Bioassay 2; mean values, *n* = 4. Bars with the same letter are not significantly different (*p* = 0.05)

In Bioassay 2, there were significant effects of insecticide (*F*_*4,30*_ = 47.16; *p* < 0.001), PB (*F*_*1,30*_ = 179.58; *p* < 0.001) and insecticide × PB interaction on *D. suzukii* mortality (*F*_*4,30*_ = 10.12; *p* < 0.001) (Fig. 2a). All four insecticides applied at the diluted doses increased mortality above the water control (*t*_30_ > 3.27; *p* = 003); mortality was further increased by the addition of PB to the droplets (*t*_30_ > 5.69; *p* < 0.001). PB solution alone did not affect mortality. There were significant effects of insecticide (*F*_*4,30*_ = 19.32; *p* < 0.001) and PB (*F*_*1,30*_ = 3.91; *p* = 0.011) on *D. suzukii* oviposition (Fig. 2b). Dilute doses of cyantraniliprole and spinosad alone did not reduce oviposition but dilute doses of lambda-cyhalothrin and deltamethrin both reduced oviposition (*t*_30_ > 2.05; *p* < 0.05) (Fig. 2b). Overall, the addition of PB to water and the dilute insecticide solutions reduced oviposition (*t*_30_ = 2.78; *p* = 0.009) although the individual effects were only significant for cyantraniliprole (*t*_30_ = 3.16; *p* = 0.004) and spinosad (*t*_30_ = 2.67; *p* = 0.012).

### Semi-field scale strawberry experiments

#### Semi-field scale experiment 1: strawberry

**Fig. 3:**
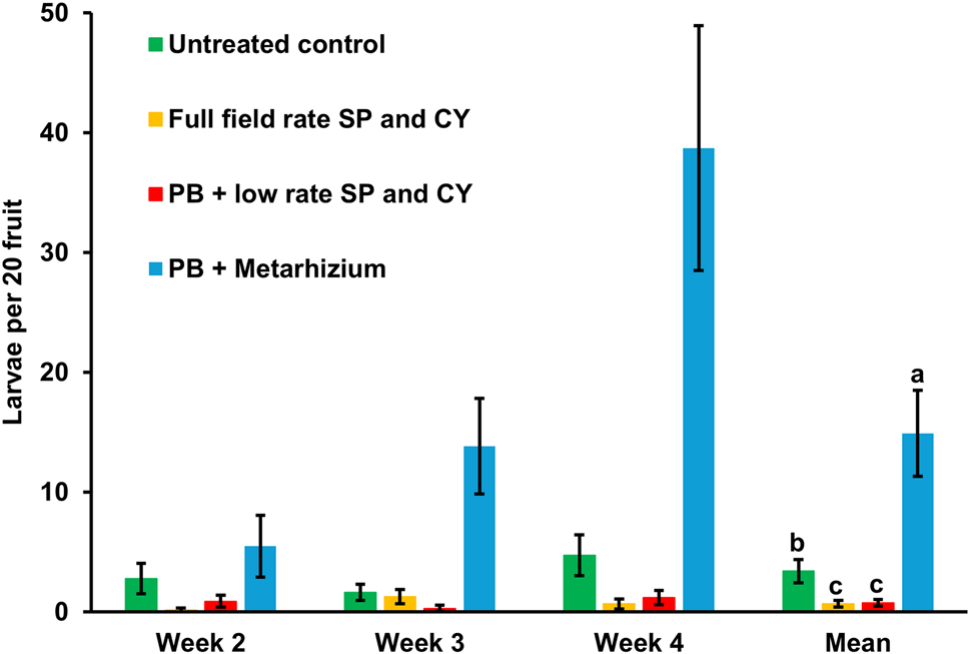
Effect of full field rates of alternating spinosad (SP) and cyantraniliprole (CY) insecticides, and low rates of the insecticides or *Metarhizium anisopliae* 35.79 with ProBandz^®^ (PB) on the number of *Drosophila suzukii* larvae extracted from fruit samples; weekly mean values ± SE, *n* = 4. Bars for overall mean values with the same letters are not significantly different (*p* = 0.05)

There were significant effects of treatment (*X*^2^_3_ = 33.24; *p* < 0.001), assessment date (*X*^2^_2_ = 12.42; *p* < 0.001) and treatment × assessment date interaction (*X*^2^_6_ = 16.63; *p* = 0.011) on the number of larvae extracted from fruit samples (Fig. 3). In the *M. anisopliae* 35.79 + PB treatment, there was a large increase in the number of larvae extracted between the second and fourth weeks of the experiment (*t* = 3.17; *p* < 0.001). There was no significant difference in larvae numbers between assessment dates for the other treatments. Averaged across assessment weeks, larvae numbers were highest in the *M. anisopliae* 35.79 + PB treatment (*t* > 3.89; *p* < 0.002) and higher in the untreated control than in the full rate insecticide or low rate insecticide + PB treatments (*t* = 3.15; *p* = 0.17). There was no significant difference between the latter two treatments.

*Metarhizium* counts in washings from leaves declined from 9.5 (± 0.5) × 10^2^ cfu ml^−1^ to 2.5 (± 0.5) × 10^2^ cfu ml^−1^ between the second and seventh days after spraying the *M. anisopliae* 35.79 inoculum suspension onto the strawberry plants. No *Metarhizium* was detected in the leaf washings from untreated strawberry plants.

#### Semi-field scale experiment 2: strawberry

**Fig. 4:**
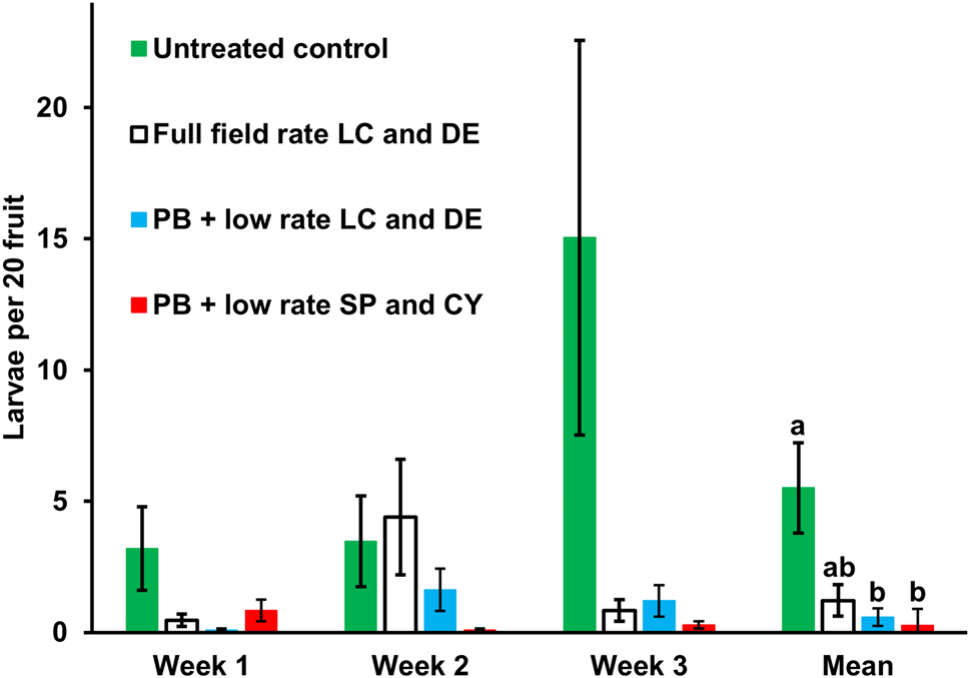
Effect of weekly sprays (starting week 0) of full field rates of alternating lambda-cyhalothrin (LC) and deltamethrin (DE), and low rates of these insecticides or alternating spinosad (SP) and cyantraniliprole (CY) with ProBandz^®^ (PB) on the number of *Drosophila suzukii* larvae extracted from fruit samples; weekly mean values ± SE, *n* = 4. Bars for overall mean values with the same letters are not significantly different (*p* = 0.05)

There was a significant effect of treatment (*G*^2^_62_ = 123.80; *p* < 0.001) on the number of larvae extracted from fruit samples (Fig. 3). The low-rate insecticide treatments + PB resulted in significantly fewer larvae than the untreated control (*z* = 3.51; *p* < 0.001) (Fig. 4). There was no significant difference between the three insecticide treatments, although the full rate spray of deltamethrin in week 2 was ineffective compared with the full rate sprays of lambda-cyhalothrin in weeks 1 and 3. No larvae flotation tests were conducted in week 4 due to insufficient fruit in the compartments although fruit from all four weeks were tested for pesticide residues.

Residues of lambda-cyhalothrin were detected in fruit sampled in weeks 1 and 3 (0.017 and 0.16 mg kg^−1^) following full rate sprays with this pesticide, and in week 4 (0.015 mg kg^−1^) following a full rate spray with deltamethrin on the same plots. No residues of deltamethrin or of any pesticides in the other three treatments were detected.

### Field scale experiments

#### Field scale experiment 1: raspberry

There was a mean of 12.0 ± 3.4 larvae per 20 fruits in raspberry samples taken for flotation tests the day before spraying commenced from both full rate insecticide (weekly alternating spinosad and cyantraniliprole) and low rate insecticide + PB allocated treatment plots. One week after the first spinosad applications, there was a mean of 6.0 ± 3.0 larvae per 20 fruits in both treatments. Larvae numbers in fruit continued to decline with subsequent cyantraniliprole and spinosad spray applications in both the full and low rate + PB treatments until no larvae were detected in fruit, 6 or 13 days after the second cyantraniliprole applications (data not shown).

#### Field scale experiment 2: raspberry

There was a mean of 33.0 ± 0.5 *D. suzukii* adults per 30 raspberry fruits in samples taken for emergence tests the day before spraying commenced from both the low rate deltamethrin + PB treatment and untreated control allocated plots. One week after spraying, the number of emerged adults from fruit samples was not significantly different from the initial samples or between treatments (37.8 ± 3.8 for low rate deltamethrin + PB treatment and 32.9 ± 6.2 for untreated control) (data not shown).

#### Field scale experiment 3: strawberry

**Fig. 5:**
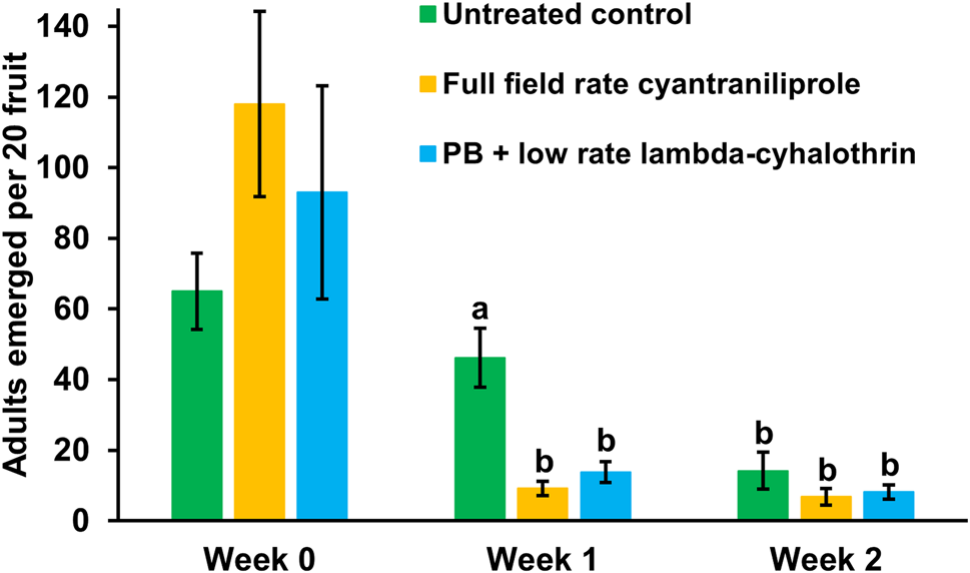
Effect of weekly sprays (starting week 0) of full field rate of cyantraniliprole or low rate of lambda-cyhalothrin with ProBandz^®^ (PB) on the number of emerged *Drosophila suzukii* adults from fruit samples; weekly mean values ± SE, *n* = 4. Bars for weeks 0, and week 1 and 2 values with the same letters are not significantly different (*p* = 0.05)

There were significant effects of spray treatment (*F*_*2,36*_ = 14.27; *p* = 0.001), sampling week (*F*_*2,36*_ = 13.11; *p* < 0.001) and treatment × sampling week interaction (*F*_*4,36*_ = 6.49; *p* = 0.006) on the number of *D. suzukii* adults that emerged from strawberry fruit samples (Fig. 5). In week 0 pre-spraying, there was a high starting population of *D. suzukii* in the tunnels, but no significant difference between the plots of allocated treatments. One week after the first sprays, there was a reduction (*t*_36_ > 5.07; *p* < 0.001) in the numbers of *D. suzukii* in fruit from the sprayed plots but no significant difference between the insecticide treatments. In week 2 samples following two sprays, the *D. suzukii* numbers had continued to decline, also in the untreated control plots (*t*_36_ > 5.02; *p* < 0.001) so that there was no significant difference with the sprayed treatments.

## Discussion

Jar bioassay results translated into semi-field scale experiments with *D. suzukii* cultures and then into field scale experiments with natural *D. suzukii* infestations although there were exceptions. *M. anisopliae* 35.79 increased mortality in jar bioassays but the effect was too slow to prevent *D. suzukii* oviposition, a drawback of EPF observed by Galland et al. (2023). Vivekanandhan et al. (2024) observed significant mortality in *D. suzukii* exposed to *M. brunneum* after five days but a constant temperature of 30 °C and high humidity were required. There was no evidence here that combining *M. anisopliae* 35.79 with PB led to increased efficacy through ingestion of the bait and EPF. Conversely, the combined use of *M. anisopliae* 35.79 and PB in a semi-field experiment led to an increase in the *D. suzukii* larvae in fruit. This was not due to attraction of *D. suzukii* adults from outside since the plots were screened. It is possible that the bait provided a food source for the female flies in the field and increased egg laying in this treatment, where the EPF was ineffective. There was no evidence from the jar bioassays that the combined use of *M. anisopliae* 35.79 and PB affected oviposition. In one week there was an 80% reduction in the *Metarhizium* inoculum on leaves in the jar bioassay and semi-field experiment which could also explain the poor efficacy.

Bacterial entomopathogens may be more effective than EPF when used in bait sprays for *D. suzukii* control since He et al. (2023) observed 100% mortality, three days following ingestion of *Bacillus cereus* H1 with sucrose solution bait. However, Hesen and van der Sluis (2017) found no *D. suzukii* control efficacy of *Bacillus thuringiensis* when used with a Combi-protec bait spray. Fanning et al. (2018) found that spraying *Chromobacterium subtsugae* reduced the number of *D. suzukii* larvae in raspberries, but control was unaffected by a corn syrup bait. However, Roubos et al. (2019) and Rhodes et al. (2023) found no efficacy of different strains of *C. subtsugae* and *Burkholderia* species when used with sucrose + yeast or ACTTRA SWD TD bait sprays respectively.

Deltamethrin in PB droplets was effective in increasing mortality and very effective in reducing oviposition in jar bioassays involving a single introduction of flies. Hesen and van der Sluis (2017) also showed *D. suzukii* control efficacy of deltamethrin in a Combi-protec bait spray in a cage experiment. However, we found that deltamethrin + PB bait sprays were ineffective in the field. This may be due to the short persistence of deltamethrin (Maguire 1990; Shaw et al. 2019a) when the bait continues to attract new cohorts of flies.

Pyrethrins applied in an ACTTRA SWD TD bait spray showed some *D. suzukii* control efficacy in laboratory and cage studies (Rhodes et al. 2023). However, pyrethrins were ineffective when applied with a Combi-protec bait in laboratory (Noble et al. 2019) and semi-field (Helsen and van der Sluis 2017) experiments, or with a corn syrup bait in raspberries (Fanning et al. 2018) and sucrose + yeast bait in blueberries (Roubos et al. 2019). Pyrethrins also have a short persistence compared with spinosad, cyantraniliprole and lambda-cyhalothrin (Lewis et al. 2016; Shaw et al. 2019a).

The higher *D. suzukii* mortality in reduced doses of lambda-cyhalothrin in bait droplets in bioassays and bait sprays in semi-field and field experiments confirms previous bioassay results (Noble et al. 2019) and cage experiments (Helsen and van der Sluis 2017). The residues of lambda-cyhalothrin on fruit following full rate sprays indicated that the pesticide persisted for at least 10 days after application, confirming the results of Shaw et al. (2019a) who demonstrated efficacy for at least 10 days after application. By contrast, no deltamethrin residues were detected in fruit samples taken immediately after spraying. Shaw et al. (2019a) also found that deltamethrin did not give effective *D. suzukii* control beyond 7 days after application.

The good *D. suzukii* control efficacy of spinosad, cyantraniliprole and lambda-cyhalothrin in full rate concentrations confirms previous results with these insecticides in laboratory bioassays (Shawer et al. 2019; Dettler et al. 2023) and in the field (Shaw et al 2019a; Shawer et al. 2019). A field raspberry experiment with natural *D. suzukii* infestations confirmed previous semi-field experiments with reared *D. suzukii* cohorts where low doses (4% of field rate) of spinosad and cyantraniliprole in bait sprays gave comparable control to full rate sprays (Noble et al. 2021, 2022). Urbaneja-Bernat et al. (2022) found that control of *D. suzukii* on commercial blueberry farms with a bait spray, HOOK SWD containing spinosad was variable in space and time. Although the bait spray reduced *D. suzukii* on some sampling dates at one farm, they were ineffective at another. Their cage experiments showed that efficacy of bait sprays declined at high fly densities but only declined slightly over a 30-day period. A more efficacious bait (ACTTRA SWD TD) containing nine attractants instead of five in HOOK SWD has since been developed (Babu et al. 2023). Comparisons of this bait with PB using a range of low dose insecticides should be conducted in field tests with a range of fruit crops. Further work is also needed to determine if PB bait sprays with low doses of lambda-cyhalothrin and other insecticides have adverse effects on non-target organisms such as the tests conducted on baits combined with spinosad (Foutain et al. 2025). If no adverse effects are found, this work will reduce dependence and risk of resistance to the two main two insecticides used for *D. suzukii* control: spinosad and cyantraniliprole.

## Author contributions

Conceptualization, R.N. and M.T.F.; methodology, A.W. S.H. and A.D.P.; formal analysis, S.H. G.D. and R.N.; investigation, B.S. R.N.; resources, A.W. and A.D.P; data curation, A.W. and A.D.P; writing—original draft preparation, R.N., A.W. and G.D.; writing—review and editing, S.H., B.S. R.N., A.W. and M.T.F.; project administration, R.N. and M.T.F; funding acquisition, R.N. and M.T.F. All authors have read and agreed to the published version of the manuscript.

## Data Availability

Requests to access the datasets should be directed to the corresponding author.
